# Cardiac rupture during the course of treatment for acute purulent pericarditis caused by *Staphylococcus aureus*: a case report

**DOI:** 10.1093/ehjcr/ytad584

**Published:** 2023-11-20

**Authors:** Ryutaro Katahira, Hiroyuki Sano, Kosuke Tanimura, Yutaka Okita

**Affiliations:** Division of Cardio-Aortic Center, Department of Internal Medicine, Takatsuki General Hospital, 1-3-13 Kosobe-cho, Takatsuki 569-1123, Japan; Division of Cardio-Aortic Center, Department of Internal Medicine, Takatsuki General Hospital, 1-3-13 Kosobe-cho, Takatsuki 569-1123, Japan; Division of Cardio-Aortic Center, Department of Internal Medicine, Takatsuki General Hospital, 1-3-13 Kosobe-cho, Takatsuki 569-1123, Japan; Division of Cardio-Aortic Center, Department of Surgery, Takatsuki General Hospital, Takatsuki, Japan

**Keywords:** Case report, Purulent pericarditis, Pseudoaneurysm, Cardiac rupture, *Staphylococcus aureus*, Echocardiography

## Abstract

**Background:**

Purulent pericarditis is rare in the modern era of antibiotics. However, it is a rapidly progressive, life-threatening disease with complications, including cardiac tamponade and left ventricular pseudoaneurysm.

**Case summary:**

A 44-year-old female was admitted with a pontine haemorrhage. On the 25th day of admission, she developed a fever along with chest pain and dyspnoea. Transthoracic echocardiography and computed tomography revealed a large pericardial effusion, leading to the diagnosis of cardiac tamponade. Pericardiocentesis was performed, resulting in the drainage of 750 mL of blood-stained fluid. Blood and pericardial fluid cultures were positive for *Staphylococcus aureus*; therefore, ceftriaxone was administered. On the 49th day, she became febrile again, and computed tomography showed increased pericardial effusion. Transthoracic echocardiography confirmed the large pericardial effusion and revealed a pseudoaneurysm on the inferior of the left ventricular wall, with blood flowing from the pseudoaneurysm into the pericardial space. Urgent surgical intervention was performed to repair a myocardial defect as a left ventricular pseudoaneurysm had ruptured in the pericardium. The patient recovered and was transferred to another hospital for rehabilitation after 108 days of hospitalization.

**Discussion:**

Purulent pericarditis can be a lethal complication; therefore, careful follow-up and strict adherence to therapeutic strategies, including the use of imaging technologies such as echocardiography, are important.

**Learning points**

- Acute purulent pericarditis caused by *Staphylococcus aureus* has a poor prognosis and requires early diagnosis, treatment, and careful observation.
- Antibiotic therapy and temporary pericardial drainage for purulent pericarditis may be therapeutically insufficient; therefore, surgical intervention may be required.
- Purulent pericarditis can be a serious and varied complication, and treatment strategies should be considered accordingly.

## Introduction

In the modern antibiotic era, purulent pericarditis is a rare but rapidly progressive and life-threatening disease. However, it is difficult to diagnose and may be easily overlooked, leading to a delayed diagnosis and the development of various complications.^1^ Early diagnosis and treatment are necessary to improve prognosis. Here, we report a rare case of cardiac rupture caused by left ventricular (LV) pseudoaneurysm, a complication of purulent pericarditis caused by *Staphylococcus aureus*.

## Summary figure

**Table:** 

Date	Event
**Before the transfer to our department**	
Day 1	A 44-year-old Japanese female with a history of hypertension but no previous treatment presented to our emergency department with right-sided paralysis and dysarthria and was admitted with a diagnosis of brainstem haemorrhage.
Day 5	She developed a fever of 37.5–38°C that lasted for several days but was diagnosed with central hyperthermia and was followed up with continued rehabilitation.
Day 15	She continued to experience fever, and ceftriaxone was initiated for urinary tract infection.
Day 22	Ceftriaxone was discontinued.
**After the transfer to our department**	
Day 25	She experienced symptoms of chest pain and dyspnoea at rest. Transthoracic echocardiogram showed massive, pericardial effusion and findings of cardiac tamponade. Therefore, the patient was transferred to our department, where pericardiocentesis was performed, resulting in the drainage of a total of 750 mL of blood-stained fluid.
Day 26	The drainage tube was removed.
Day 27	Blood and pericardial fluid cultures were positive for *S. aureus*. We started antibiotic treatment with ceftriaxone (2 g/day).
Day 28	Transoesophageal echocardiography (TEE) showed no evidence of infective endocarditis and bidirectional shunt flow through patent foramen ovale.
Day 33	Computed tomography (CT) showed no significant change in residual pericardial fluid.
Day 49	Transthoracic echocardiography (TTE) revealed a pseudoaneurysm on the inferior LV wall, and cardiac rupture with ongoing purulent pericarditis was suspected. Urgent surgical intervention was performed.
Day 59	Diuretics were initiated to manage right heart failure.
Day 70	Antibiotic treatment was discontinued, and she continued rehabilitation with concomitant treatment of total parenteral nutrition.
Day 108	The patient was transferred elsewhere for rehabilitation.

## Case presentation

**Figure 1:**
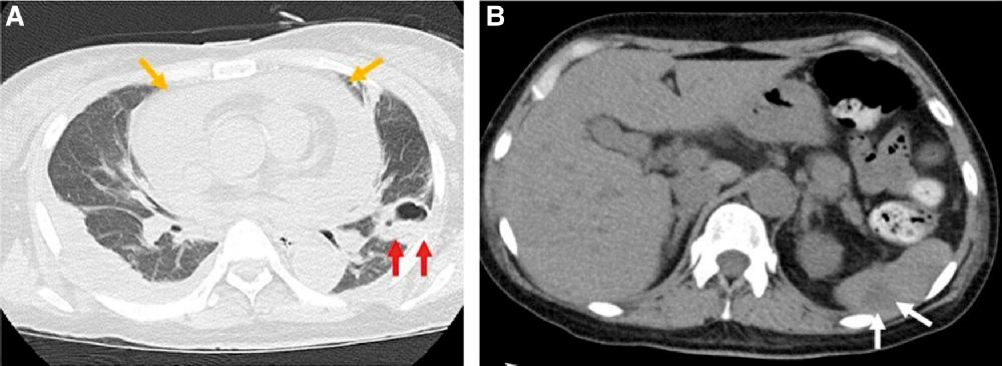
Computed tomography images of the chest at admission. Plain computed tomography of the chest shows bilateral pleural effusion, large pericardial effusion (orange arrows), splenic infarction (white arrows), and consolidation and cavity lesions in the upper-left lung zones (red arrows) (*A* and *B*).

**Figure 2:**
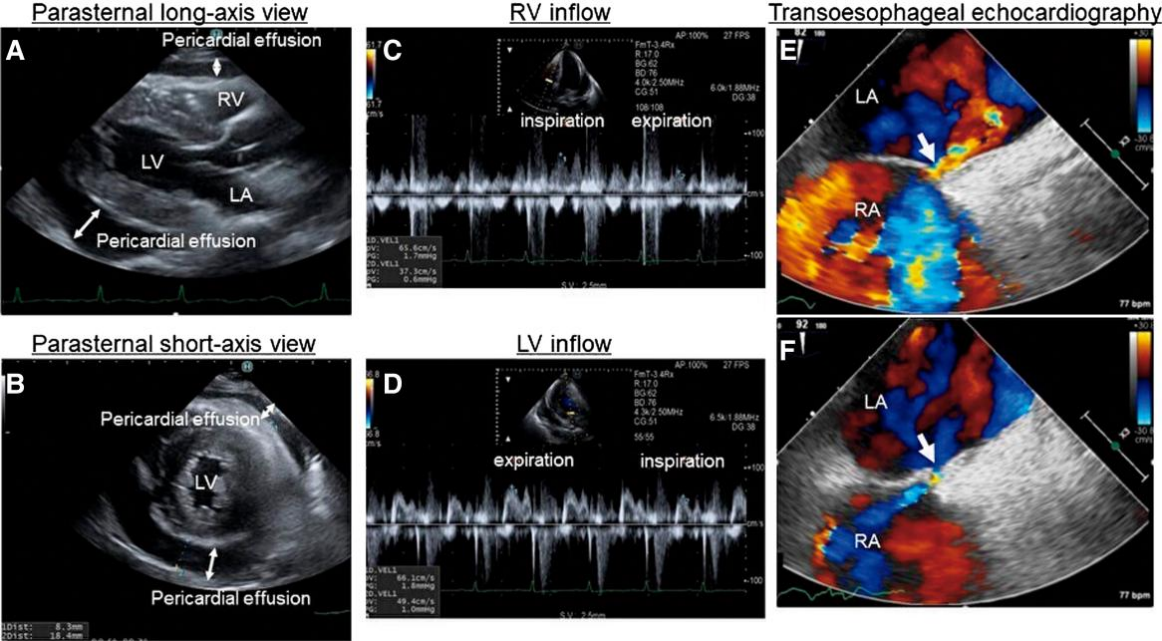
Transthoracic echocardiography images. Transthoracic echocardiography (parasternal long-axis and short-axis views) shows a large circumferential pericardial effusion (*A* and *B*). A pulsed-wave Doppler echocardiogram shows a 25% decrease in the E-wave velocity of the left ventricular inflow during inspiration, while the E-wave velocity of the right ventricular inflow increased by 76% during inspiration (*C* and *D*). Transoesophageal echocardiography shows no evidence of infective endocarditis and bidirectional shunt flow through patent foramen ovale (*E* and *F*). LA, left atrium; LV, left ventricular; RV, right ventricular.

**Figure 3:**
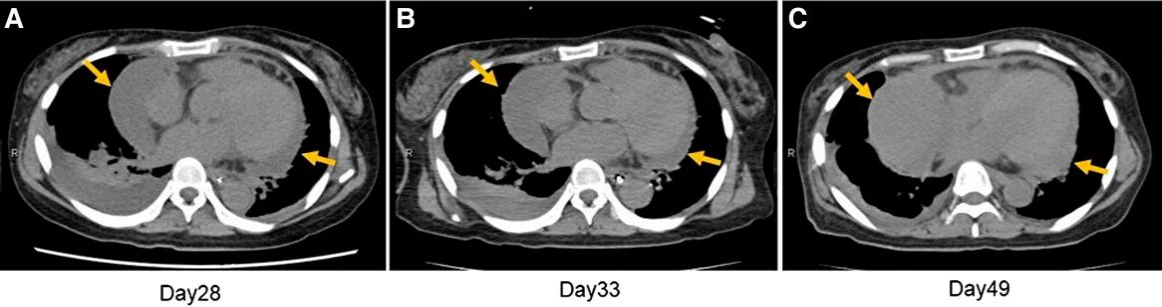
Computed tomography images after pericardiocentesis. After pericardiocentesis, computed tomography on Day 28 of admission shows residual pericardial fluid in the lateral posterior left ventricular wall and right atrial side (orange arrow) (*A*). On Day 33 of admission, computed tomography shows no significant change of residual pericardial fluid (*B*). On Day 49 of admission, computed tomography also shows a further increase in pericardial effusion (*C*).

**Figure 4:**
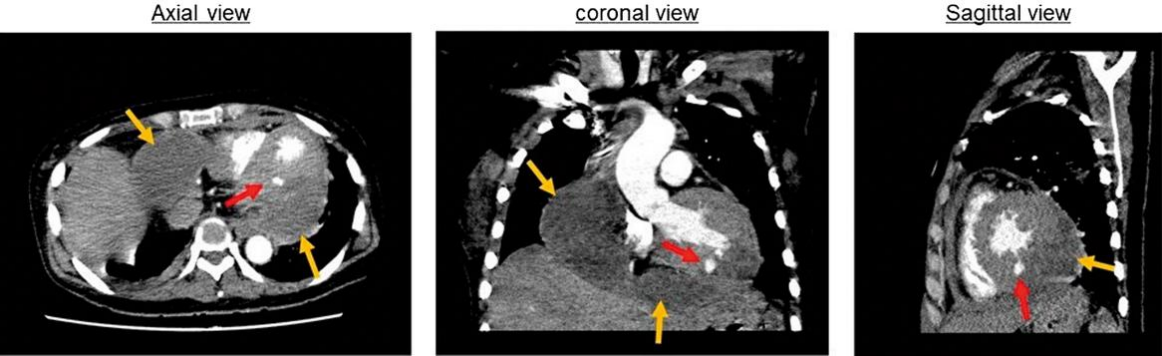
Contrast-enhanced computed tomography images of the chest. Contrast-enhanced computed tomography of the chest shows a large pericardial effusion of moderate density (orange arrows) and pseudoaneurysm on the inferior left ventricular wall (red arrows).

**Figure 5:**
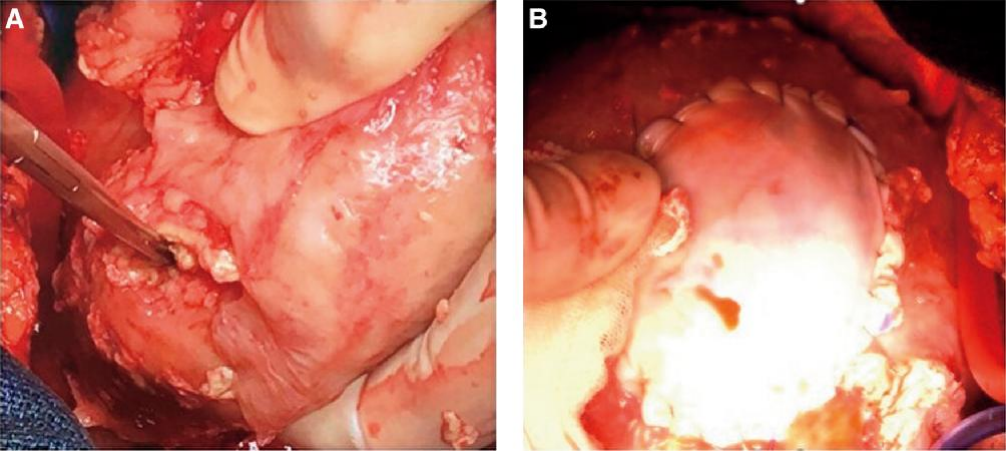
Myocardial repair. A 2 cm-sized defect and thinning of the myocardium are seen at the posterior left ventricular wall (*A*). For myocardial reconstruction, the left ventricular perforation is repaired using a 5 cm large bovine pericardium soaked in glutaraldehyde (*B*).

A 44-year-old Japanese female with a medical history of untreated hypertension presented to our emergency department with right-side paralysis and dysarthria and was admitted with a diagnosis of pontine haemorrhage. On the fifth day after admission, she developed a fever (37.5–38°C) that lasted for several days but was subsequently diagnosed with central hyperthermia. On the 15th day, ceftriaxone (2 g/day) was administrated for urinary tract infection; nevertheless, on the 25th day, she developed chest pain and dyspnoea. Physical examination revealed the following: blood pressure, 94/45 mmHg; pulse rate, 102 b.p.m. (regular); and oxygen saturation, 96% (O_2_ 1 L/min). The jugular venous pressure was observed to be elevated; however, no cardiac murmur, abnormal lung sounds, or pericardial rub were detected. The electrocardiogram showed normal sinus rhythm and ST-segment elevation (1 mm) in the inferior leads. Chest radiography showed cardiomegaly and bilateral pleural effusion. The white blood cell count (WBC) was 10 000/mm (reference, 3300–8600/mm). Further biochemical analysis revealed the following: no evidence of elevated myocardial enzymes, C-reactive protein, 7.4 mg/dL (reference, 0–0.14 mg/dL); N-terminal pro-B-type natriuretic peptide, 8138 pg/mL (reference, ≤55 pg/mL); and creatinine, 3.38 mg/dL (reference, 0.65–1.07 mg/dL). Plain chest CT showed bilateral pleural effusions, large pericardial effusion, splenic infarction, and consolidation and cavity lesions in the upper left lung zones (*Figure 1*). Transthoracic echocardiography revealed normal biventricular function but a large circumferential pericardial effusion, inferior vena cava dilation, and right ventricular (RV) collapse. Pulsed-wave Doppler echocardiogram showed a 25% decrease in the E-wave velocity of the LV inflow during inspiration, while the E-wave velocity of the RV inflow increased by 76% during inspiration, which was determined to be the signs of cardiac tamponade (*Figure 2*). The patient was referred to our department, where an emergency pericardiocentesis was performed for suspected cardiac tamponade due to acute pericarditis. A pericardial drainage tube was placed, obtaining 750 mL of blood-stained fluid. Subsequent TTE showed mild residual pericardial fluid in the posterior LV wall; however, the drain was removed 24 h later due to haemodynamic improvement and minimal output. Pericardial fluid cytometry revealed an elevated count of inflammatory cells with no malignant cells. Blood and pericardial fluid cultures were positive for *S. aureus*, sensitive to ceftriaxone. Transoesophageal echocardiography showed no evidence of infective endocarditis and bidirectional shunt flow through patent foramen ovale (*Figure 2*). Considering these results, acute purulent pericarditis due to hospital-acquired pneumonia caused by *S. aureus* infection was diagnosed. The patient was treated with intravenous ceftriaxone (2 g/day); nonetheless, on the 49th day after admission, she became febrile again and had elevated inflammatory markers (WBC, 13 000/mm; C-reactive protein, 6.97 mg/dL). Computed tomography showed increased pericardial effusion (*Figure 3*). Transthoracic echocardiography revealed dyskinesis and a pseudoaneurysm on the inferior LV wall and a large pericardial effusion located behind the inferior LV wall (see Supplementary material online, *Video S1*), with blood flow from the pseudoaneurysm into the pericardial space (see Supplementary material online, *Videos S2* and *S3*). Contrast-enhanced chest CT showed a large, moderately dense pericardial effusion and a pseudoaneurysm on the inferior LV wall (*Figure 4*). Emergency coronary angiography performed to identify ischaemic heart disease did not show stenosis or any evidence of myocardial infarction. Preoperative TEE also showed a pseudoaneurysm in the inferior LV wall, a large pericardial effusion behind the inferior LV wall, and blood flow from the pseudoaneurysm into the pericardial space (see Supplementary material online, *Video S4*). Cardiac rupture with ongoing purulent pericarditis was suspected, and urgent surgical intervention was conducted. Preoperative cerebral angiography revealed no abnormal findings. After consultation with the neurosurgeon, anticoagulation therapy was administered as usual during surgery. After sternotomy, the tense pericardium was incised, and bloody pericardial fluid was detected. A cardiopulmonary bypass was established, and the ascending aorta was clamped. A 2 cm-sized myocardial defect and myocardial thinning had developed at the posterior LV wall. The defect was closed using a 5 cm large glutaraldehyde-treated bovine pericardial patch (*Figure 5*). Ceftriaxone was continued for 3 weeks postoperatively. Diuretic therapy (torasemide, 2 mg/day) was required to manage right heart failure caused by moderate or more severe tricuspid regurgitation, and the patient’s condition was stable. She was transferred to a rehabilitation hospital after 108 days of hospitalization. Approximately 18 months post-surgery, subsequent clinical and echocardiographic examinations indicated a stable patient condition.

## Discussion

Although purulent pericarditis is rare and its occurrence is declining in the modern antibiotic era, it is a life-threatening disease.^1^ The mortality rate of purulent pericarditis caused by *S. aureus* is as high as 40% because of complications including cardiac tamponade, LV pseudoaneurysm, and myocardial abscess.^2–4^ Additionally, *S. aureus* is the most common pathogen causing purulent pericarditis,^1,5^ and its potent toxicity causes haematogenous spread and cardiac structural destruction, leading to a rapid clinical course of exacerbation. Left ventricular pseudoaneurysms are a very rare complication of purulent pericarditis. Juliana *et al*.^2^ reported that most cases complicated by LV pseudoaneurysm were caused by *S. aureus* and occurred at the LV posterior and/or lateral wall. Left ventricular pseudoaneurysms have an approximately 30–45% risk of rupture if left untreated.^6^ Although the exact mechanism by which purulent pericarditis complicates LV pseudoaneurysm is unknown, it is believed to be associated with direct seeding of infected pericardial fluid, causing myocardial injury. In our patient’s clinical course, pericardial effusion remained after pericardiocentesis, and despite continued antimicrobial therapy, the infected pericardial effusion may have caused inflammation and myocardial damage. Purulent effusions can rapidly re-accumulate, and only one-third of cases of purulent pericarditis can be managed by pericardiocentesis alone; most cases require surgical pericardiectomy or other procedures.^1,7^ Therefore, intrapericardial thrombolysis and subxiphoid pericardiotomy may have been necessary to prevent pseudoaneurysm and cardiac rupture in our case. However, TTE performed in this case indicated that the sub-endocardium of LV may have been damaged first, spreading to the epicardium to form LV pseudoaneurysm. Another conceivable pathogenesis of LV pseudoaneurysm is septic thromboembolism caused by *S. aureus* in the coronary artery, resulting in myocardial injury.^1^ Furthermore, TEE showed evidence of bidirectional shunt flow through patent foramen ovale in this case. Therefore, we assumed that the pathological mechanism was a septic paradoxical embolism in the coronary arteries. Cardiac magnetic resonance imaging (MRI) is often useful in confirming sub-endocardial or transmural infarction. However, in this case, cardiac MRI was not performed perioperatively because blood investigations revealed no evidence of elevated myocardial enzymes, and the bedside TTE showed no abnormal LV wall motion until cardiac rupture. Furthermore, late gadolinium-enhanced cardiac MRI could not be performed as the patient had an estimated glomerular filtration rate of <30 mL/min/1.73 m^2^.

Purulent pericarditis requires thorough scrutiny for an accurate diagnosis and poses serious complications, requiring early diagnosis and careful monitoring. The possible occurrence of constrictive pericarditis in the course of purulent pericarditis has also been reported to be at least 3.5% and as high as 20–30% of cases.^8,9^ Transthoracic echocardiography is convenient and useful, and close haemodynamic and echocardiographic monitoring during treatment could improve prognosis.

## Supplementary Material

ytad584_Supplementary_Data

## Data Availability

The data underlying this article are available in the article and in its online supplementary material.

## Lead author biography

Ryutaro Katahira went into the path of cardiology after 2 years of residency. After 3 years of working as a general cardiologist, he is now a cardiologist at Takatsuki General Hospital.
